# The trends in prostate specific antigen usage amongst United Kingdom urologists – a questionnaire based study

**DOI:** 10.1186/1471-2490-8-17

**Published:** 2008-11-20

**Authors:** Helena P Burden, Chris R Davis, Sophie Tate, Raj Persad, Chris H Holmes, Kate Whittington

**Affiliations:** 1 Urology Speciality Registrar, Derriford, Plymouth, UK; 2 Foundation 1, Southmead Hospital, Westbury-upon-Trym, Bristol, BS10 5NB, UK; 3 Faculty of Medical and Veterinary Sciences, University of Bristol, School of Medical Sciences, University Walk, Bristol, BS8 1TD, UK; 4 Urology Department, Bristol Royal Infirmary, Marlborough Street, Bristol, BS2 8HW, UK; 5 Clinical Sciences South Bristol, St. Michael's Hospital, Southwell Street, Bristol, BS2 8EG, UK; 6 Clinical Sciences South Bristol, Dorothy Hodgkin Building, Whitson Street, Bristol, BS1 3NY, UK

## Abstract

**Background:**

Worldwide, the use of prostate specific antigen (PSA) testing as a screen for prostate cancer is contentious. Whilst there is no National UK Screening programme, many men undergo opportunistic screening. This study investigates UK urologist's usage of PSA and the awareness surrounding the Department of Health (DoH) PSA guidelines.

**Methods:**

Urologists were sent a questionnaire regarding PSA cut-off values.

**Results:**

Of the 733 urologists eligible to participate in this study 346 returned completed questionnaires giving a response rate of 47%. The most commonly generally used age-related PSA cut-off values (36% of respondents) are – 3.5 ng/ml for 50 – 59 year olds, 4.5 ng/ml for 60 – 69 year olds and 6.5 ng/ml for over 70 year olds. Two-thirds (58%, 200/346) of respondents were aware of the DoH PSA guidelines but only 20% (n = 69/346) follow these guidelines. The majority of respondents (68%, n = 234/346) used higher PSA cut-offs than recommended by the DoH. The level of compliance showed marked regional variation with a range from 7% to 44% (median 19%). In addition, it was apparent that lower PSA cut-off values were used in private practice as opposed to the National Health Service.

**Conclusion:**

A nationwide lack of agreement on PSA cut-off values may generate a variable standard of care both regionally and in NHS versus private practice. Generally, higher PSA cut-off values are being used than recommended by the DoH guidance.

## Background

Prostate cancer is the most common male cancer, with nearly 32,000 men diagnosed in the UK each year [1]. Unlike breast and cervical cancer, its female counterparts, there is no national United Kingdom (UK) screening programme for prostate cancer. However at present opportunistic screening is occurring, with many physicians using the serum Prostate-Specific Antigen (PSA) test.

PSA, also known as human kallikrein 2, is produced by the luminal prostatic cells which line prostatic acini. PSA is responsible for liquefying semen and hence has a major role in fertility [2]. It is possible to detect PSA in small amounts in the serum of healthy males and this level increases in prostate cancer [3]. However, whilst PSA is tissue specific an increase in circulating levels is not definitively linked to tumour development. Urethral instrumentation, urinary infection, prostatitis, urinary retention, ejaculation and benign prostatic hypertrophy all raise serum PSA levels [3]. The PSA serum test was initially used as a sign of recurrent disease following radical prostatectomy or radiotherapy for prostate cancer, but came into mainstream usage as an diagnostic test in the 1980s [4].

Based upon a cut-off level of 4 ng/ml, the standard serum PSA test has a sensitivity of approximately 90% and a specificity of approximately 40% [5]. However, these values change depending upon the specified PSA cut-off level. The high false positive rate leads to a large number of men undergoing further investigations, which are unnecessary, invasive and raise anxiety. False negative tests also occur and serve to give false reassurance, as some prostate cancer will be missed.

There is no worldwide consensus on prostate cancer screening. There are several studies to date that have looked at the benefit of screening for prostate cancer. An observational study from Tyrol, Austria [6] reported on the geographical differences observed between Tyrol, where PSA testing had been freely available since 1993, and the rest of Austria, where it hadn't. The study showed a marked decline in prostate cancer mortality detected in Tyrol as compared to the rest of Austria. The European Randomised Study of Screening for Prostate Cancer (ERSPC) is still underway, however preliminary results suggest that screen detected prostate cancer has more favourable prognostic indicators that non-screen detected prostate cancer, but as yet no mortality advantage to screening has been shown [7].

The USA is the only country currently to have a national prostate cancer screening programme. The American Cancer Society and American Urological Association recommend an annual PSA test for all men aged 50 or over with an estimated 10 year life expectancy or more [8]. A PSA cut-off value of 4.0 ng/ml is used [9].

Despite not having a National Screening Programme in the UK, the Department of Health has released guidelines in the form of the Prostate Cancer Risk Management Programme for General Practitioners involved in using the PSA test [10]. The Prostate Cancer Risk Management Programme was produced by the National Health Service (NHS) Cancer Screening Programme and Cancer Research UK under advice from a multi-disciplinary expert group set up by the Department of Health. This recommended that men concerned about prostate cancer should be offered a PSA test but only after fully informed consent following discussion of the limitations of the test. The PSA cut-off levels that they suggest, either for referral to a urologist or for consideration of further investigation, are shown in Table 1.

This study was therefore designed to investigate the usage of PSA cut-off values amongst Urologists in the UK and to determine the awareness of the Department of Health PSA guidelines. This was investigated in Consultants working in both the National Health Service (the free UK Government led service) and/or the private sector.

## Methods

### Study design

We conducted a questionnaire-based study on the usage of PSA cut-off values. Self-administered questionnaires (see additional file 1) were sent to urologists across England, Wales and Northern Ireland. Urologists in Scotland were excluded from this study as they have a separate Department of Health.

Two rounds of questionnaires were sent to the same group of urologists, the same exclusion criteria (outlined in detail below) were used for both rounds. Questionnaires were initially e-mailed to potential participants and non-responders were sent a subsequent postal and repeat e-mail questionnaire.

In the first round we contacted 961 urologists thought eligible to take part in this study – of these 178 initially replied (response rate = 19%). In the second round a further 228 urologists were found to be ineligible for the study based upon exclusion criteria outlined below. This left a total of 733 urologists eligible to take part – 178 had originally replied and a further 168 replied in the second round, thus giving a total number of 346 urologists who replied. Response rate was therefore increased to 346/733 = 47%.

The primary outcome measures were levels of the PSA reference values used and awareness of the Department of Health PSA guidelines. Secondary outcome measures included the use of additional PSA-linked investigations and an opinion on the current guidelines and implementation of a national PSA screening programme.

Ethical approval was not needed for this study.

### Participants

733 urologists were identified from the British Association of Urologists (BAUS) Members Handbook as being eligible for this study based upon their membership criteria. Only full members, junior members and associate urologist specialist members were included in this study. Specific exclusion criteria included senior membership of BAUS, overseas membership, un-contactable urologists (e.g. those not providing an address), and urologists not currently participating in oncological practice (e.g. paediatric urologists).

### Statistical analysis

Data has been analysed using Graphpad Prism version 5.0 software USA, statistical analysis used the unpaired t test and Fishers exact.

## Results

### Characteristics of respondents

Of the 733 urologists eligible to participate in this study 346 returned completed questionnaires giving a response rate of 47%. The majority of respondents were consultants (193/346 – 56%) with the remaining 44% being composed of registrars and non-consultant career grades. Of the 346 respondents 202 (58%) treated NHS patients only, 2 (0.01%) treated private patients only and 142 (41%) treated both patient groups. Participants were divided into 10 regions, the number of responses per region were: Eastern (A) – 26, London (B) – 62, North West (C) – 41, Northern and Yorkshire (D) – 42, South East (E) – 33, South West (F) – 50, Trent (G) – 27, West Midlands (H) – 35, Wales (I) – 12, and Northern Ireland (J) – 18.

### Usage of age-related PSA cut-off values

**Figure 1:**
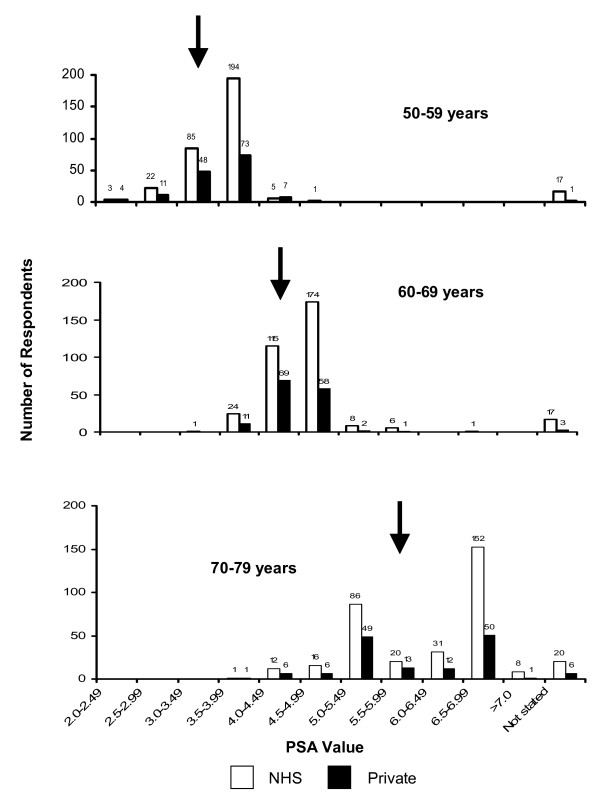
**Illustration of range of age-specific prostate specific antigen cut-off values and the number of respondents using them**. Black arrows  indicate Department of Health guidelines.

In each of the three age groups a wide range of PSA reference values were being used (50–59 year olds: 2.0–5.0 ng/ml; 60–69 year olds: 3.0–6.5 ng/ml; 70–79 year olds: 3.5–7.5 ng/ml). The most commonly used (36% of respondents) set of age-related PSA cut-off values was 2.5 ng/ml for 40 – 49 year olds, 3.5 ng/ml for 50 – 59 year olds, 4.5 ng/ml for 60 – 69 year olds and 6.5 ng/ml for 70–79 year olds. The Department of Health uses age-related PSA cut-offs in its guidelines, as shown in Table 1. 20% (n = 69/346) of respondents followed the Department of Health PSA age-related cut-off values in their current clinical practice. Age-matched PSA cut-off values for NHS versus private practice were compared, showing 20% (68/344) vs 26% (38/144), respectively, of urologists using the DoH PSA guidelines. When this was further investigated it was clear that most respondents, in both NHS and private practice, were using values higher than those recommended by the Department of Health (Figure 1). The median PSA cut-off values for ages 60–69 and 70–79 were higher in NHS (4.5 and 6.0 ng/ml respectively) compared to private (4.0 and 5.5, respectively) practice (Table 1). When direct comparison of NHS and private age-related PSA reference values was made for the 142 respondents who saw both groups of patients we found that 10% (n = 14/142) used lower age-related reference values in their private versus their NHS practice.

**Figure 2:**
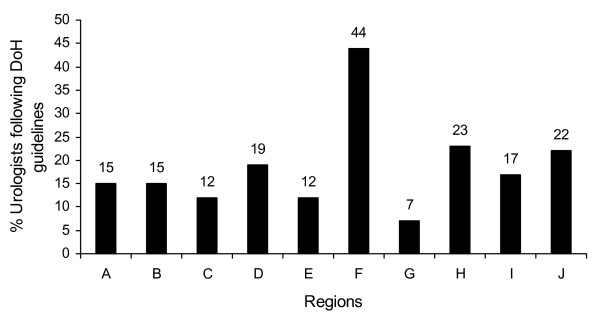
**Percentage of urologists using Department of Health prostate specific antigen guidelines.** Split by region (A - J).

Interestingly, there were clear regional differences across the UK (Figure 2). In the regions, between 7% (2/27 – region G) and 44% (22/50 – region F) of Urologists were following the DoH PSA guidelines.

### Use of PSA-Related Tests

Other PSA-related test procedures, such as PSA density, velocity, ratios of free to total PSA and analysis of PSA isoforms, have been suggested to enhance the sensitivity and clinical relevance of the standard serum PSA test. The standard serum PSA test alone was used by one in five (18%, n = 62/144) of respondents for NHS practice, whilst one in three (35%, n = 51/144) of respondents relied solely on this test for private practice. The most common additional test used by respondents was PSA velocity, which was used by 75% (n = 255/342) of respondents in NHS practice and 82% (n = 118/144) of respondents in their private practice (Table 2).

### Awareness of, and agreement with the Department of Health Guidelines

Despite the widespread use of PSA testing in urological practice and the ready availability of the Department of Health PSA Guidelines on the internet  and elsewhere, less than two-thirds (58%, 200/346) of respondents were aware of the recommended PSA cut-off values. Of those respondents who were aware of the guidelines: 57% (114/200) agreed with the values cited; 24% (47/200) disagreed; 12% (24/200) offered no opinion, while 7% did not respond to the question.

A majority of respondents (59%, n = 205/346) believe that there should not be a National screening programme for prostate cancer based upon the Department of Health serum PSA guidelines. The most common reason for this, cited by 30 of the 81 (37%) urologists commenting on this in the free text response box provided, was a lack of evidence-based medicine supporting the case for PSA screening.

## Discussion

The principal finding of this study is that, in the UK, there is a widespread variation in the application of PSA cut-off values among urologists, which may be leading to an inequality in men's healthcare related to PSA testing. Although just under two-thirds of urologists participating in this study were aware of the Department of Health PSA age-related guidelines, less than a third used them in everyday clinical practice. Interestingly, research has shown that only 56% of General Practitioner (GP) partners are aware of the PSA guidelines [11].

Whilst these guidelines were intended for GPs, they have also been sent to many urologists within the United Kingdom. The PSA guidelines were formed because of the dilemma faced by all medical staff dealing with asymptomatic men who request or have a PSA test, in that there are many pitfalls associated with the sensitivity and specificity of the PSA test. For this reason there is no screening programme within the UK. However opportunistic testing occurs both in general practice and within secondary care by urologists and other physicians. Whilst further research is awaited the government has attempted to guide practice in a practical manner via these PSA guidelines.

It is important to raise awareness of how the PSA test should be correctly utilised not just amongst GPs, but amongst all of the medical profession.

The transition of patients between primary care and secondary care via referral pathways is much more efficient if both Urologists and GPs are aware of the referral guidelines. Therefore for this point alone it is important for urologists to be aware of the DoH PSA guidelines.

Once a patient arrives in the secondary care setting after being referred with an abnormal PSA result a decision must be made as to whether the patient needs to undergo a prostate biopsy. When making this decision, the urologist takes not only the PSA result into account, but also urological symptoms, family history and digital rectal examination. This is a complex decision, involving discussion with the patient regarding the sensitivity and specificity of a PSA test, together with morbidity involved in a prostate biopsy. However, when making the final decision of when to biopsy – whether the PSA is within the "normal" levels, and what these levels are perceived to be plays a large role. Many laboratories within the UK are using the DoH referral guidelines as PSA cut-off values, which will in turn be used by urologists as a guide to normality levels, and thus will aid in the decision making process of whether to biopsy a man.

This lack of compliance with the Department of Health PSA guidelines is likely to be due to the paucity of evidence supporting the recommended age-related PSA cut-offs, and this was commented on by a number of participants.

The only published report suggesting PSA age-related cut-offs is a study by Oesterling et al. in 1993 [12]. This population-based study in Olmstead County, USA, recruited 2119 men between the ages of 40–79, to investigate the natural history of benign prostatic hyperplasia. A quarter of these men were randomly chosen to undergo a PSA test, a digital rectal examination and a Trans-Rectal Ultrasound (TRUS) guided prostate biopsy. PSA values for the 88% of men with no evidence of prostate cancer were evaluated. On this basis, PSA cut-offs were suggested of 2.5 ng/ml for 40 – 49 year olds, 3.5 ng/ml for 50 – 59 year olds, 4.5 ng/ml for 60 – 69 year olds and 6.5 ng/ml for 70–79 year olds based upon the serum PSA 95^th ^percentiles. Interestingly this was the most widely used set of PSA cut-offs in our study, being used by 36% of respondents.

Wide regional differences in the age-related PSA cut-off values may potentially lead to subsequent regional variation in the likelihood of men undergoing further invasive investigations, such as TRUS guided prostatic biopsies. In addition, when PSA cut-off values are compared between urologists treating patients in the NHS and the private sector it is clear that lower values are being used in private healthcare. Hence patients being treated by the NHS are less likely to have further investigations which may potentially result in the later detection of prostate cancer in a small percentage of these patients.

An interesting observation from our study is the wide range of PSA cut-off values being used for each age group. By using data from the Prostate Cancer Prevention Trial (PCPT) placebo group [13], we are able to extrapolate the percentage likelihood of a man being diagnosed with prostate cancer as indicated by their PSA value. For example, in our study a man in the 50 – 59 year age group has a PSA cut-off value ranging from 2.0 to 5.0 ng/ml. Using PCPT data, a PSA cut-off of 5.0 ng/ml rather than 2.0 ng/ml more than doubles the likelihood of having prostate cancer detected at biopsy. Perhaps more worryingly, in the PCPT study the risk of detecting high grade prostate cancers increases threefold (5.7% to 14.6%) when a PSA cut-off of 5.0 ng/ml rather than 2.0 ng/ml is used. Therefore, the use of higher PSA cut-off values may result in men with high grade prostate cancers being detected later.

The response rate for this study was 47% which is comparable to other questionnaire based studies of urologists. Survey-based studies have the inherent problem of selection bias and the resultant effect on conclusions is difficult to determine. Any comparisons that are made in our results may well be affected by the lack of response from 53% of urologists, and bias may well have been introduced because of this. Particularly in the comparison between regions, as relatively small numbers are being compared, and we are unable to assess where the 53% of urologists who didn't reply are based.

However, it could be argued that the individuals who are more likely to participate in a survey such as this are the enthusiastic group of urologists who are more likely to be aware of the existence of Department of Health guidelines and current practices involving PSA cut-offs. Therefore this would suggest that the group who didn't reply actually may have more variable use of age-related PSA cut-offs than our study has quoted.

Interestingly Meyer et al. [14] investigated the uniformity of prostate cancer guidelines by comparing the guidelines provided by the British Association of Urological Surgeons, European Association of Urology and American Urological Association and also found that there was a general lack of uniformity in the management of prostate cancer.

There remain several unanswered questions regarding the natural history of, and best treatment for, prostate cancer and a number of large trials investigating such questions are underway (The United States Prostate, Lung, Colon and Ovary Screening Trial, The European Randomised Study of Screening for Prostate Cancer and The United Kingdom PROstate Testing for Cancer and Treatment study). The results of such trials are likely to initiate discussion regarding prostate cancer screening and the role of PSA in this. An important implication of the results presented here is that they highlight a lack of discussion, both within the urological community and between urologists and the Department of Health, regarding current PSA cut-off guidelines. We hope that avenues will be explored to improve discussion and hence obtain widespread agreement on any future PSA cut-off guidelines. UK men have a right to receive a standardised level of care throughout the country.

## Conclusion

The principal finding is that most urologists surveyed use evidence-based cut-offs, which however are not in accordance with the guidelines suggested by the DoH. A significant proportion of urologists surveyed appear to be unaware of the DoH guidelines. There is, therefore, a wide range of age related PSA cut-offs being used throughout the United Kingdom, and differences are also apparent between the levels used in the NHS and private practice. Thus creating an inequality in the healthcare received by men throughout the country.

## Competing interests

The authors declare that they have no competing interests.

## Authors' contributions

All authors contributed to conception of the idea. HB/CD performed the data collection. HB drafted the initial manuscript. All authors critically revised the manuscript and have given final approval for this version to be published.

## Pre-publication history

The pre-publication history for this paper can be accessed here:



## Supplementary Material

Additional file 1 **Questionnaire for appendix.** Questionnaire used in study. Click here for file
