# Supplementary material for: The trends in prostate specific antigen usage amongst United Kingdom urologists – a questionnaire based study
Source: BMC Urol. 2008 Nov 20;8:17. doi: 10.1186/1471-2490-8-17 (PMC2606676; doi:10.1186/1471-2490-8-17)
Supplement: Additional file 1 — Questionnaire for appendix. Questionnaire used in study. [file 1471-2490-8-17-S1.doc]

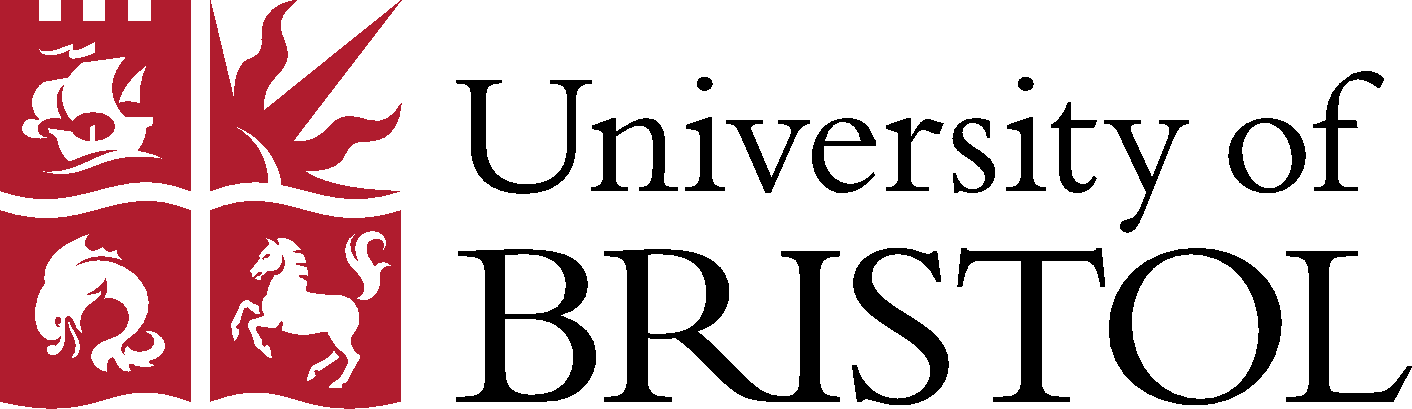
**Serum Prostate Specific Antigen (PSA) Test Questionnaire**

Thank you for taking the time to complete this brief questionnaire into Hospital Guidelines and the clinical use of PSA in asymptomatic men with no known prostate disease.

**Please write an X beside the appropriate options below**:

1. **I am a:**

Consultant:

Non-consultant career-grade:

Specialist Registrar:

Research Registrar:

Senior House Officer:

Other (please state) ……………………………in Urology

1. **What region are you currently working in?**

Eastern:

London:

North West:

Northern and Yorkshire:

South East:

South West:

Trent:

West Midlands:

Other (Please state)………………………………………

1. **Please state your Hospital’s guidelines for age-related PSA cut-offs:**

**NHS:** **Private:**

| Age | PSA Cut-off (ng/mL) |
| --- | --- |
| 50-59 |  |
| 60-69 |  |
| ≥70 |  |

| Age | PSA Cut-off (ng/mL) |
| --- | --- |
| 50-59 |  |
| 60-69 |  |
| ≥70 |  |

**Or if using different age ranges please fill in the table below:**

**NHS:** **Private:**

| Age | PSA Cut-off (ng/mL) |
| --- | --- |
|  |  |
|  |  |
|  |  |
|  |  |
|  |  |

| Age | PSA Cut-off (ng/mL) |
| --- | --- |
|  |  |
|  |  |
|  |  |
|  |  |
|  |  |

1. **Are you aware of the Department of Health Prostate Cancer Risk Management Programme PSA age-range guidelines?**

Yes:

No:

1. **Do you use the Department of Health PSA guidelines in your own clinical practice?**

**NHS:** Yes:

No:

**Private:** Yes:

No:

1. **If No are there any other Guidelines that you do use?**

NHS ……………………………………………………………...

Private ……………………………………………………………...

1. **Do you agree with the limits given by the DOH?**

Yes:

No:

Don’t know DOH limits:

1. **If No what limits do you think should be used instead?**

…………………………………………………………………………

…………………………………………………………………………

1. **What other measures of PSA are you using routinely in your clinical practice?**

**NHS:**

PSA density:

PSA velocity:

PSA ratio free:total:

PSA isoforms:

Other: (Please state)…………………………………………………

**Private:**

PSA density:

PSA velocity:

PSA ratio free:total:

PSA isoforms:

Other: (Please state)…………………………………………………

1. **Do you think the Department of Health PSA age-related cut-offs should be used as part of a National Screening programme?**

**(State reasons if you wish)**

Yes:

No:

………………………………………………………………………………
